# Chronic Kidney Failure Provokes the Enrichment of Terminally Differentiated CD8^+^ T Cells, Impairing Cytotoxic Mechanisms After Kidney Transplantation

**DOI:** 10.3389/fimmu.2022.752570

**Published:** 2022-05-03

**Authors:** Jonas Leonhard, Matthias Schaier, Florian Kälble, Volker Eckstein, Martin Zeier, Andrea Steinborn

**Affiliations:** ^1^ Department of Obstetrics and Gynecology, University of Heidelberg, Heidelberg, Germany; ^2^ Department of Nephrology, University of Heidelberg, Heidelberg, Germany; ^3^ Department of Internal Medicine V (Hematology), University of Heidelberg, Heidelberg, Germany

**Keywords:** chronic kidney failure, dialysis treatment, kidney transplantation, CD8^+^ T cell differentiation, CD8^+^ regulatory T cells, cytotoxic mechanisms, CD8^+^ TEMRA cells

## Abstract

Chronic kidney failure (KF) provokes the development of immune senescent CD8^+^ cytotoxic T cells, affecting the occurrence of graft rejection, viral infections, and malignancies after kidney transplantation. In this study, we analyzed the impact of KF, subsequent dialysis treatment, and kidney transplantation on the differentiation of CD8^+^CD31^+^CD45RA^+^CCR7^+^ recent thymic emigrant (CCR7^+^ RTE) Tregs/Tresps into CD8^+^CD31^-^CD45RA^-^ memory (CD31^-^ memory) Tregs/Tresps and its effect on the release of cytokines, Fas receptor, Fas ligand as well as cytotoxic mediators by naïve, central memory (CM), effector memory (EM), and terminally differentiated effector memory (TEMRA) Tresps. We found that normal age-dependent differentiation of CD8^+^ Tregs/Tresps generally differs in the way that TEMRA cells only arise in Tresps. Compared to healthy controls, KF patients revealed an age-independently decreased frequency of CCR7^+^ RTE Tregs/Tresps, but increased frequencies of CCR7^+^ MN Tregs/Tresps and CD31^-^ memory Tregs/Tresps, suggesting an increased differentiation *via* CD31^+^CD45RA^-^ memory (CD31^+^ memory) Tregs/Tresps into CD31^-^ memory Tregs/Tresps. Intensified differentiation *via* CD31^+^ memory Tresps increased the emergence of apoptosis-resistant CM Tresps with strong Fas ligand-mediated cytotoxicity. CCR7^+^ RTE Tresp proliferation generated TEMRA Tresps, secreting high levels of cytotoxic mediators. In dialysis and transplant patients, CD31^+^ TEMRA Tregs/Tresps accumulated, proposing an impaired CCR7^+^ RTE Treg/Tresp differentiation *via* CD31^+^ memory Tregs/Tresps into CD31^-^ memory Tregs/Tresps. Increased percentages of CD31^-^ TEMRA Tresps, but not of CD31^-^ TEMRA Tregs, were observed in all patient groups, indicating impaired proliferation of CCR7^+^ RTE Tresps, but not of CCR7^+^ RTE Tregs, into CD31^-^ memory Tregs/Tresps. In transplant patients, CCR7^+^ RTE Tregs accumulated, while frequencies of CCR7^+^ RTE Tresps were decreased, suggesting that the immunosuppressive therapy only prevented excessive CCR7^+^ RTE Treg differentiation but not that of CCR7^+^ RTE Tresps. Presumably, this caused the accumulation of TEMRA Tresps with decreased release of cytotoxic mediators, such as perforin. In conclusion, we propose that chronic KF affects both the differentiation of CD8^+^ Tregs and CD8^+^ Tresps. However, the immunosuppressive therapy after transplantation may successfully prevent excessive Treg differentiation, but not as suffciently that of Tresps. Therefore, the risk for graft rejection may be reduced, while the susceptibility for infections and malignancies may be increased in these patients.

## Introduction

Chronic kidney disease (CKD) is characterized by the continuous loss of kidney function over months to years. Depending on the severity of symptoms, kidney replacement therapy must be considered at the final stage of CKD, called kidney failure (KF). It was shown that uremic toxins are causally responsible for immune dysfunctions (1–3), particularly affecting the T cell compartment (4, 5). Lower thymic output and thus an increased differentiation of the already distributed T cells provoke accelerated aging of the T cells, even affecting survival after kidney transplantation (6–9). This is already known for CD4^+^ T helper cells, which coordinate cellular and humoral immunity, and CD8^+^ cytotoxic T cells, which eliminate cells infected with intracellular pathogens and tumor cells (10–13). Both responder T cells (Tresps) and regulatory T cells (Tregs), which play a crucial role in the suppression of activated Tresps, seem to undergo increased differentiation and consequently experience advanced exhaustion (14). This immune dysfunction cannot be restored with kidney replacement therapy (15) and leads to a decreased immune response to infections, malignancies, and vaccinations in these patients (16–18). Otherwise, low-grade activated T cells cause chronic inflammation and thereby favor cardiovascular disease (19). Kidney transplantation is the preferred kidney replacement therapy since it reduces mortality and improves long-term survival compared to dialysis treatment (20, 21).

While changes in the differentiation of CD4^+^ T cells were shown to affect their Treg/Tresp ratio and thus their functionality in dialysis and transplant patients (14, 22), the impact of kidney replacement therapies on the differentiation and functionality of CD8^+^ cytotoxic T cells is far less studied. Research is required as CD8^+^ T cells play a crucial role in the occurrence of post-transplant cutaneous squamous cell carcinoma and kidney allograft rejection (23–25). CD8^+^ Tregs are getting increased interest as it is planned to use these cells to prevent rejection in a phase I cell therapy study in kidney transplant patients due to their high suppressive capacity (26).

Two different approaches to investigate the differentiation of CD8^+^ T cells may be applied. Chemokine receptor type 7 (CCR7, CD197) and CD45RA are common surface markers to examine the differentiation of CCR7^+^CD45RA^+^ naïve cells into CCR7^+^CD45RA^-^ central memory (CM) cells, CCR7^-^CD45RA^-^ effector memory (EM) cells, and CCR7^-^CD45RA^+^ terminally differentiated effector memory (TEMRA) cells. In the course of this, CMs as precursor effector cells are thought to possess immune-stimulatory functions, while EMs and TEMRAs, as finally differentiated cells, exert effector functions (27). Otherwise, the differentiation of CD45RA^+^CD31^+^ recent thymic emigrants (RTEs) into CD45RA^+^CD31^-^ mature naïve (MN) cells, CD45RA^-^CD31^+^ memory (CD31^+^ memory) cells, and CD45RA^-^CD31^-^ memory (CD31^-^ memory) cells may be of importance to ascertain progressive immune senescence. Thereby, MN T cells arise by post-thymic RTE proliferation and maintain the naïve T cell pool throughout life as a long-living reserve population (28). These cells may preserve differentiation in case of RTE exhaustion. Here we combined both approaches so that unexperienced CD31^+^CD45RA^+^CCR7^+^ RTEs (CCR7^+^ RTEs) may differentiate into CD31^-^CD45RA^+^CCR7^+^ resting - MNs (CCR7^+^ MNs), CD31^+^CD45RA^+^CCR7^-^ TEMRAs (CD31^+^ TEMRAs), CD31^-^CD45RA^+^CCR7^-^ TEMRAs (CD31^-^TEMRAs), CD31^+^ memory T cells, and CD31^-^ memory T cells. CD31^+^ and CD31^-^ TEMRAs may represent T cells, which have reached their maximum differentiation capacity at an earlier stage of differentiation.

In this study, we investigated the impact of KF followed by replacement therapies, such as dialysis and kidney transplantation, on CD8^+^ Treg/Tresp differentiation and examined whether differences in the differentiation of these cells affected the Treg/Tresp ratio or influenced the functionality of CD8^+^ Tresps. We found decreased frequencies of naive CCR7^+^ RTE Tregs/Tresps, but increased frequencies of more mature cell populations, such as CM, EM, or TEMRA Tregs/Tresps, proposing an increased CD8^+^ T cell differentiation in KF and dialysis patients. In kidney transplant patients, the accumulation of CCR7^+^ RTE Tregs, but decreased percentages of both CD31^+^ and CD31^-^ memory Tregs within total CD8^+^ Tregs, suggests that the immunosuppressive therapy effectively suppresses excessive CD8^+^ Treg differentiation. In contrast, the frequency of CCR7^+^ RTE Tresps was decreased, while that of CD31^+^ and CD31^-^ TEMRA Tresps was increased, suggesting an increased differentiation into TEMRA Tresps concomitant with altered cytotoxic activity, such as reduced perforin release. Thereby, a severely diminished CD8^+^ Treg/Tresp ratio was favored in kidney transplant patients. In conclusion, excessive differentiation of CD8^+^ Tregs may not progress after transplantation, whereas increased Tresp differentiation may continue, altering cytotoxic activity.

## Materials and Methods

### Patient Collectives and Healthy Controls

For quantitative analysis, peripheral venous blood samples were collected from 134 healthy volunteers, 57 KF patients without replacement therapy (KF patients), 87 KF patients treated with dialysis (dialysis patients), and 138 kidney transplant recipients. Exclusion criteria for healthy controls were recent illness within the previous 3 months, general disease, elevated C-reactive protein (CRP), or serum creatinine levels above 1 mg/dl. Blood samples from KF patients were obtained during hospitalization immediately before the initiation of dialysis therapy. Dialysis patients had undergone treatment for at least 3 months. For these patients, recent illness during the last 3 months, significant anemia (hemoglobin < 8 g/dl), cancer, and administration of immunosuppressive drugs were evaluated as an exclusion criterion. Blood samples were collected at the beginning of the repeated hemodialysis, or concerning peritoneal dialysis patients, during routine visits. Kidney transplant recipients were included in the study if the transplantation had occurred at least 3 months before and if patients did not have recent illness, autoimmune disease, or cancer. To avoid falsification by infections or rejection periods, a CRP > 5 mg/l (21 patients) and serum creatinine > 2 mg/dl (9 patients) were criteria for exclusion. Blood samples were collected during routine visits at the Department of Nephrology, University of Heidelberg. **Table 1** reveals the clinical data of all participants.

**Table 1:** Clinical characteristics of the study participants.

	Healthy controls n = 134	KF patients n = 57	Dialysis patients n = 87	Transplant patients n = 138
Female sex, n (%)	84 (63%)	17 (32%)	23 (26%)	58 (42%)
Age (years)	50 (22-88)	61 (21-84)	58 (23-90)	52 (23-82)
Primary disease, n (%)	−			
Diabetes mellitus		13 (23%)	15 (17%)	4 (3%)
Hypertension		6 (11%)	13 (15%)	12 (9%)
GN/vasculitis		14 (25%)	21 (24%)	62 (45%)
Interstitial nephritis		4 (7%)	2 (2%)	4 (3%)
Polycystic kidney disease		7 (12%)	11 (13%)	24 (17%)
Cardio-renal syndrome		4 (7%)	2 (2%)	0 (0%)
Obstructive uropathy		0 (0%)	3 (3%)	12 (9%)
Others		5 (9%)	11 (13%)	10 (7%)
Unknown		4 (7%)	9 (10%)	10 (7%)
Dialysis method	−	−		
HD, n (%)			57 (66%)	66 (48%)
CAPD, n (%)			24 (28%)	25 (18%)
Both, n (%)			6 (7%)	17 (12%)
None, n (%)			−	23 (17%)
Time on dialysis (y)	−	−	3.0 (0.2-39.7)	2.6 (0-12.8)
Deceased donor kidney, n (%)	−	−	−	71 (51%)
Time since transplantation (y)	−	−	−	6.2 (0.2-28.4)
Immunosuppression	−	−	−	
Tac + MPA + Steroid				47 (34%)
CsA + MPA + Steroid				57 (41%)
mTOR-inh. + MPA + Steroid				7 (5%)
Azathioprine + others				5 (4%)
Belatacept + others				2 (1%)
Others				20 (14%)
Serum creatinine (mg/dl)	<1.0	5.72 (2.24-14.05)	7.96 (2.43-17.23)	1.26 (0.63-1.90)
Urea (mg/dl)	<40	149 (67-298)	109 (24-194)	40 (14-84)
CKD-EPI GFR (ml/min/1.73m²)	>90	9.0 (4.0-28.6)	6.2 (2.2-25)	60.8 (32.3-114.8)
C-reactive protein (mg/l)	<5	4.6 (<2.0-106.5)	3.0 (<2.0-34.3)	<2.0 (<2.0-4.7)

The data are presented as their median values together with their range (minimum-maximum). CAPD, continuous ambulatory peritoneal dialysis; CKD-EPI GFR, chronic kidney disease epidemiology collaboration estimated glomerular filtration rate; GN, glomerulonephritis; HD, hemodialysis; KF, kidney failure; MPA, mycophenolic acid; mTOR-inh., mechanistic target of rapamycin-inhibitor; Tac, tacrolimus.

The Regional Ethics Committee approved the study (reference number S-523/2012, 05.07.2018). All participants were fully informed about the study settings and aims. Written informed consent was received from all patients and volunteers. Nomenclature is based on the current Kidney Disease: Improving Global Outcomes (KDIGO) Consensus Conference recommendation (29).

### Fluorescence-Activated Cell Sorting Staining

Nine ml of venous blood from all participants were collected into ethylenediaminetetraacetic acid (EDTA)-containing tubes. Peripheral blood mononuclear cells (PBMCs) were isolated *via* density gradient centrifugation using Lymphodex (Inno-Train Diagnostik GmbH, Kronberg, Germany). PBMCs (8 x 10^6^) in a volume of 100 µl were surface-stained with 10 µl peridinin-chlorophyll-protein (PerCP)-conjugated anti-CD8 (BD Biosciences, Heidelberg, Germany), 5 µl phycoerythrin-cyanine 7 (PE-Cy7)-conjugated anti-CD127 (eBioscience, Frankfurt, Germany), 5 µl phycoerythrin (PE)-conjugated anti-CCR7 (Biolegend, San Diego, USA), 5 µl allophycocyanin-H7 (APC-H7)-conjugated anti-CD45RA (BD Biosciences) and 5 µl Alexa Fluor 647-conjugated anti-CD31 (BD Biosciences) undiluted mouse monoclonal antibodies. After 20 minutes, PBMCs were washed twice with 3 ml phosphate-buffered saline (PBS) and centrifugalized with 483 g for 5 minutes. Intracellular forkhead box P3 (FoxP3) was detected by using a fluorescein isothiocyanate (FITC)-conjugated anti-human FoxP3 staining set (clone PCH101, eBioscience) according to the manufacturer’s instructions. Negative control samples were incubated with isotype-matched antibodies. A FACSCanto cytometer (BD Biosciences) was used for six-color flow cytometric analysis. Dead cells and doublets were excluded *via* forward and side scatter characteristics (FSC and SSC). Statistical analysis was based on at least 100.000 CD8^+^ T cells.

### Gating Strategy, Cell Types, and Differentiation Pathways

**Figure 1:**
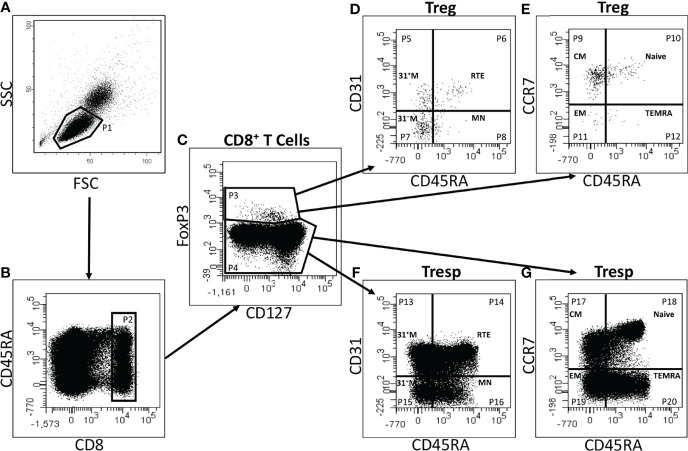
Gating strategy for six-color-flow-cytometric detection of CD8^+^CD127^low+/-^FoxP3^+^ Tregs, CD8^+^CD127^+/-^FoxP3^-^ Tresps and their subsets. At first, lymphocytes (P1) were identified by side scatter characteristics (SSC) versus forward scatter characteristics (FSC) **(A)**. The percentage of CD8^+^ T cells (P2) was estimated by analyzing the fluorescence activity of CD8 versus CD45RA **(B)**. To separate Tregs (P3) from Tresps (P4), fluorescence activity of FoxP3 versus CD127 was presented **(C)**. The percentages of RTE Tregs/Tresps (P6, P14), MN Tregs/Tresps (P8, P16), CD31^+^ memory Tregs/Tresps (P5, P13), and CD31^-^ memory Tregs/Tresps (P7, P15) were determined by analyzing the fluorescence activity of CD31 versus CD45RA, respectively **(D, F)**. Naïve Tregs/Tresps (P10, P18), CM Tregs/Tresps (P9, P17), EM Tregs/Tresps (P11, P19), and TEMRA Tregs/Tresps (P12, P20) were identified by estimating the fluorescence activity of CCR7 versus CD45RA **(E, G)**.

**Figure 1** exhibits the gating strategy for the quantitative analysis. We determined the percentages of total CD8^+^ T cells of all lymphocytes as well as their percentages of CD8^+^ Tregs and CD8^+^ Tresps. Afterwards, we determined the composition of the total CD8^+^ Treg/Tresp pool with naïve, CM, EM, and TEMRA cells during the course of life. To recognize differences in the differentiation of CD8^+^ Tregs/Tresps between healthy controls and the study groups, the two differentiation schemata (naïve, CMs, EMs, TEMRAs versus RTEs, MNs, CD31^+^ memory cells, CD31^-^ memory cells) were merged to distinguish newly released antigen-unexperienced CCR7^+^ RTE Tregs/Tresps and CCR7^+^ resting-MN Tregs/Tresps from terminally differentiated CD31^+^ TEMRA Tregs/Tresps and CD31^-^ TEMRA Tregs/Tresps. Finally, we divided the total CD8^+^ Treg/Tresp pool into the six following subsets: CCR7^+^ RTE, CCR7^+^ resting-MN, CD31^+^ TEMRA, CD31^-^ TEMRA, CD31^+^ memory, and CD31^-^ memory Tregs/Tresps. By this procedure, different differentiation pathways of CCR7^+^ RTE Tregs/Tresps were identified:

(1) *via* differentiation into CD31^+^ memory Tregs/Tresps and subsequent proliferation into CD31^-^ memory Tregs/Tresps,
(2) *via* direct proliferation of CCR7^+^ RTE Tregs/Tresps into CD31^-^ memory Tregs/Tresps, and
(3) *via* proliferation into CCR7^+^ resting-MN Tregs/Tresps and subsequent differentiation into CD31^-^ memory Tregs/Tresps.

Thereby, we assume that CD31^+^ TEMRA Tregs/Tresps arise from an impaired differentiation of CCR7^+^ RTE Tregs/Tresps *via* CD31^+^ memory Tregs/Tresps, while CD31^-^ TEMRA Tregs/Tresps emerge from impaired CCR7^+^ RTE Treg/Tresp proliferation.

### Positive Selection of CD8^+^ T Cells

For the analysis of cytokine secretion, 45 ml venous blood samples were obtained in EDTA-containing tubes from 10 individuals of each control and study group (KF, dialysis, and kidney transplant patients). **Table 2** summarizes the clinical data of these participants. Ficoll-Hypaque (Inno-Train Diagnostik GmbH) density gradient centrifugation was used to isolate the PBMCs. Then, CD8^+^ T cells were selected *via* magnetic-activated cell sorting (MACS) using the CD8^+^ T cell Isolation Kit (Miltenyi Biotec, Bergisch Gladbach, Germany) according to the manufacturer’s instructions. The purity of the isolated CD8^+^ T cells was 86.7% +/- 10.2%.

**Table 2:** Clinical characteristics for the functional CD8^+^ Tresp analysis.

	Healthy controls n = 10	KF patients n = 10	Dialysis patients n = 10	Transplant patients n = 10
Female sex, n (%)	4 (40%)	4 (40%)	4 (40%)	3 (30%)
Age (years)	56 (26-60)	51 (29-69)	56 (27-72)	56 (35-68)
Primary disease, n (%)	−			
Diabetes mellitus		2 (20%)	3 (30%)	0 (0%)
Hypertension		0 (0%)	0 (0%)	2 (20%)
GN/vasculitis		6 (60%)	2 (20%)	6 (60%)
Interstitial nephritis		0 (0%)	2 (20%)	0 (0%)
Polycystic kidney disease		2 (20%)	1 (10%)	1 (10%)
Obstructive uropathy		0 (0%)	2 (20%)	0 (0%)
Unknown		0 (0%)	0 (0%)	1 (10%)
Dialysis method	−	−		
HD, n (%)			7 (70%)	6 (60%)
CAPD, n (%)			3 (30%)	2 (20%)
None, n (%)			−	2 (20%)
Time on dialysis (y)	−	−	2.2 (0.3-12.0)	3.6 (0-10.9)
Deceased donor kidney, n (%)	−	−	−	5 (50%)
Time since transplantation (y)	−	−	−	7.3 (1.3-20.0)
Immunosuppression	−	−	−	
Tac + MPA + Steroid				6 (60%)
CsA + MPA + Steroid				4 (40%)
Serum creatinine (mg/dl)	<1.0	6.19 (4.07-15.32)	8.97 (4.19-12.01)	1.30 (0.84-1.68)
Urea (mg/dl)	<40	164 (104-255)	108 (83-142)	51 (19-66)
CKD-EPI GFR (ml/min/1.73m²)	>90	8.1 (3.7-11.4)	6.0 (3.9-11.3)	53.1 (42.0-107.2)
C-reactive protein (mg/l)	<5	2.4 (<2.0-35.5)	2.0 (<2.0-13.9)	<2.0 (<2.0-3.9)

The data are presented as their median values together with their range (minimum-maximum). CAPD, continuous ambulatory peritoneal dialysis; CKD-EPI GFR, chronic kidney disease epidemiology collaboration estimated glomerular filtration rate; GN, glomerulonephritis; HD, hemodialysis; KF, kidney failure; MPA, mycophenolic acid; Tac, tacrolimus.

### Sorting and Bead-Based Immunoassay of the Different CD8^+^ Tresp Subsets

After purification, CD8^+^ T cells in a volume of 100 µl were surface-stained with 10 µl PerCP-conjugated anti-CD8 (BD Biosciences), 5 µl PE-Cy7-conjugated anti-CD127 (eBioscience), 20 µl PE-conjugated anti-CD25 (BD Biosciences), 5 µl APC-H7-conjugated anti-CD45RA (BD Biosciences) and 5 µl BD Horizon V450-conjugated anti-CCR7 (BD Biosciences) undiluted mouse monoclonal antibodies for 20 minutes, washed twice with 3 ml phosphate-buffered saline (PBS) and centrifugalized with 483 g for 5 minutes. In all experiments, FSC and SSC were used to recognize dead cells and doublets. Afterward, CD8^+^ T cells were isolated by depleting CD8^+^CD25^+^CD127^low+/-^ Tregs and CD8^+^CD25^-^CD127^+/-^ Tresps were sorted into CCR7^+^CD45RA^+^ naïve, CCR7^+^CD45RA^-^ CM, CCR7^-^CD45RA^-^ EM, and CCR7^-^CD45RA^+^ TEMRA Tresps using a FACSAria sorter (BD Biosciences). Subsequently, 4 x 10^4^ cells of each of these Tresp subsets were cultured in 100 µl X-VIVO15 medium (Lonza, Verviers, Belgium), supplemented with 1 µl/ml soluble anti-CD3 and 2 µl/ml soluble anti-CD28 antibodies (eBioscience) to stimulate cytokine secretion. Incubation conditions were 37 °C temperature and 5 % CO_2_ concentration. Supernatants were harvested after 72 h and frozen at -80 °C. After thawing, the LEGENDplex Human CD8/NK Mix and Match Subpanel (13-plex, Biolegend) was used for the measurement of granulysin, granzyme A, interferon-γ (IFN-γ), interleukin-2 (IL-2), perforin, soluble Fas receptor (sFAS-R), soluble Fas ligand (sFAS-L) and tumor necrosis factor-alpha (TNF-α) in the supernatants according to the manufacturer’s instructions. Bead-based analysis was performed with a FACSCanto cytometer (BD Biosciences). Data analysis was achieved with the LEGENDplex Data Analysis Software V8.0 (Biolegend).

### Statistical Analysis

Linear regression was used to examine changes in the composition of CD8^+^ T cells with Tregs and Tresps, as well as changes in the Treg/Tresp ratio, during the course of life using separate models for all patient groups. The same approach was applied to the percentages of the Treg/Tresp subsets within the total Treg/Tresp pool to recognize changes with age. Differences between healthy volunteers and each study group concerning CD8^+^ T cells, Tregs, Tresps, the Treg/Tresp ratio, and their subsets were examined using multiple regression analysis adjusted for the age variable (centered on the mean), wherein an interaction term of the age and the patient group was included. Differences in the concentrations of each analyte between the four Tresp subsets in healthy controls were determined with the non-parametric Friedmann test followed by the Bonferroni-Holm *post hoc* test. Changes between the age-matched control and the respective study group were detected with the non-parametric Wilcoxon-Mann-Whitney U-test. A p-value < 0.05 was considered significant. However, this research is an exploratory study in which the calculated p-values are purely descriptive, but not confirmatory. For all tests, the software package BiAS for Windows (version 10.06) was used.

## Results

### Severely Diminished CD8^+^ Treg/Tresp Ratio in Kidney Transplant Patients

**Figure 2:**
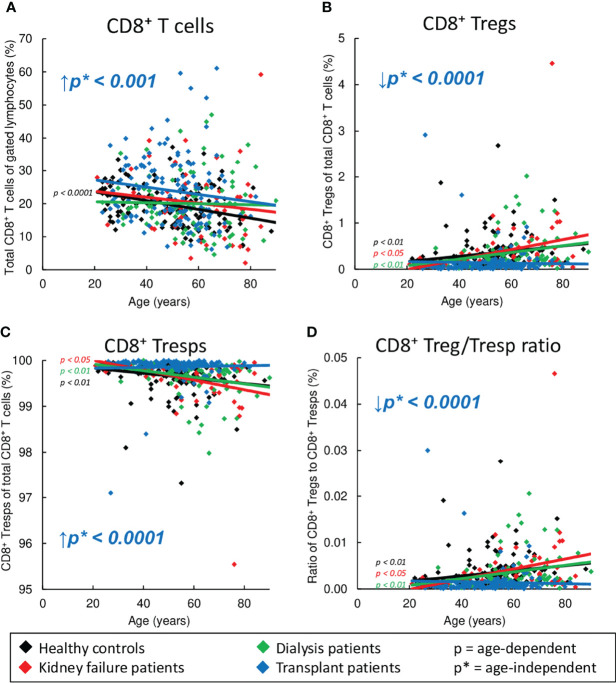
Changes in the composition of CD8^+^ T cells with Tregs and Tresps in healthy controls (n=134), KF patients (n=57), dialysis patients (n=87), and kidney transplant patients (n=138). The diagrams show the percentages of CD8^+^ T cells of all lymphocytes **(A)**, CD8^+^CD127^low+/-^FoxP3^+^ Tregs **(B)** and CD8^+^CD127^+/-^FoxP3^-^ Tresps **(C)** within total CD8^+^ T cells as well as the CD8^+^ Treg/Tresp ratio **(D)** in healthy volunteers (♦), KF patients (♦), dialysis patients (♦), and kidney transplant patients (♦). Regression lines concerning changes in the percentages with age are shown for both healthy volunteers and all patient groups. Significant changes are marked by color-matched p-values. Age-independent significant differences between healthy volunteers and study groups are marked by an arrow (↑↓) and their color-matched p*-values.

In healthy volunteers, we found a significant decrease of CD8^+^ T cells (**Figure 2A**) with an increase of CD8^+^ Tregs and a decline of CD8^+^ Tresps (**Figures 2B, C**), causing a significant increase in the Treg/Tresp ratio with age (**Figure 2D**). Similar findings were obtained for KF and dialysis patients, proposing that normal age-dependent differentiation of CD8^+^ Tregs/Tresps is largely preserved. However, kidney transplant recipients did not show an increase in Tregs and decrease in Tresps and thus no improvement in the Treg/Tresp ratio during the course of life (**Figures 2B–D**). Moreover, compared to healthy volunteers, the percentage of total CD8^+^ T cells was age-independently increased in kidney transplant patients with a significantly decreased percentage of Tregs but an increased percentage of Tresps, causing a significant shift in the Treg/Tresp ratio in favor of Tresps (**Figures 2A–D**). These results imply substantial changes in the differentiation of both CD8^+^ Tregs and Tresps in kidney transplant patients compared to healthy controls.

### Strong Accumulation of Naïve Tregs, but Not of Naïve Tresps in Kidney Transplant Patients

**Figure 3:**
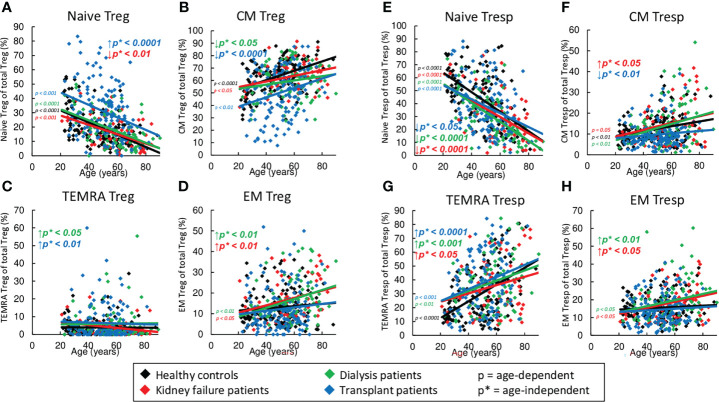
Differentiation of CD8^+^ Tregs and CD8^+^ Tresps in healthy controls (n=134), KF patients (n=57), dialysis patients (n=87), and kidney transplant patients (n=138). The figures present the percentages of naïve **(A, E)**, CM **(B, F)**, TEMRA **(C, G)**, and EM Tregs/Tresps **(D, H)** within total Tregs/Tresps of healthy volunteers (♦), KF patients (♦), dialysis patients (♦), and kidney transplant patients (♦). Regression lines concerning changes with age are shown for both healthy volunteers and all patient groups. Significant changes are marked by color-matched p-values. Age-independent significant differences between healthy volunteers and study groups are marked by an arrow (↑↓) and their color-matched p*-values.

Similar to healthy volunteers, naïve Tregs decreased significantly with age in all three patient groups, while CM and EM Tregs tended to increase (**Figures 3A, B, D**). Neither healthy volunteers nor patients within study groups showed an age-dependent increase of TEMRA Tregs (**Figure 3C**). Regardless of age, compared to healthy controls, KF and dialysis patients showed an enrichment mainly of EM Tregs (**Figure 3D**). In transplant patients, the frequency of naïve Tregs was strongly increased (**Figure 3A**), while that of CM Tregs was markedly decreased (**Figure 3B**). Both dialysis and transplant patients revealed a significant accumulation of TEMRA Tregs (**Figure 3C**).

Age-dependent shifts in the Tresp subsets were found to be similar compared to those of Tregs, namely naïve Tresps decreased significantly, while CM and EM Tresps increased (**Figures 3E, F, H**). However, in all groups there was an additional increase of TEMRA Tresps with age (**Figure 3G**). Regardless of age, all patient groups revealed a strong decrease of naïve Tresps and an increase of TEMRA Tresps compared to healthy volunteers (**Figures 3E, G**). Therefore, additional increased frequencies of CM and EM Tresps were detected in KF patients (**Figures 3F, H**), while dialysis patients showed an enrichment of EM and TEMRA Tresps only (**Figures 3G, H**). In transplant patients, decreased frequencies of naïve Tresps were detected within the total CD8^+^ Tresp pool (**Figure 3E**) in contrast to increased frequencies of naïve Tregs within the CD8^+^ Treg pool (**Figure 3A**). Similar to Tregs, decreased frequencies of CM Tresps (**Figure 3F**) but increased frequencies of TEMRA Tresps (**Figure 3G**) were detected.

### Markedly Reduced Frequency of CD31^+^ and CD31^-^ Memory Tregs in Kidney Transplant Patients

**Figure 4:**
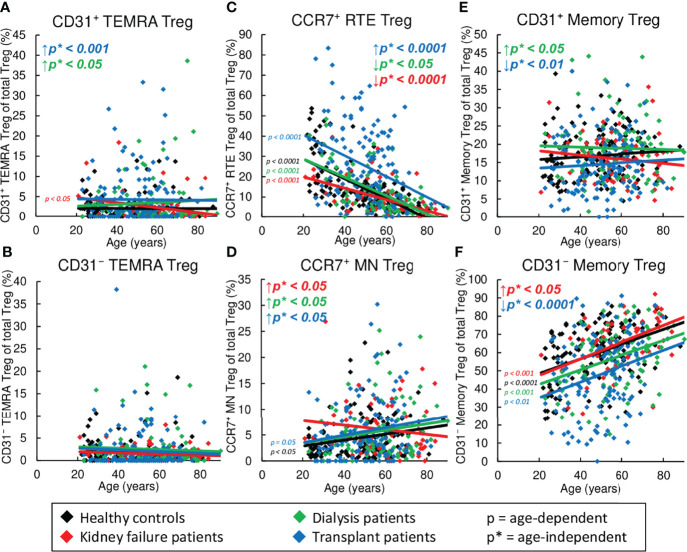
Changes in the composition of CD8^+^ Tregs with differentially matured subsets in healthy controls (n=134), KF patients (n=57), dialysis patients (n=87), and kidney transplant patients (n=138). The figures present the percentages of CD31^+^ TEMRA **(A)**, CD31^-^ TEMRA **(B)**, CCR7^+^ RTE **(C)**, CCR7^+^ MN **(D)**, CD31^+^ memory **(E)**, and CD31^-^ memory Tregs **(F)** within total Tregs of healthy volunteers (♦), KF patients (♦), dialysis patients (♦), and kidney transplant patients (♦). Regression lines concerning changes in the percentages with age are shown for both healthy volunteers and all patient groups. Significant changes are marked by color-matched p-values. Age-independent significant differences between healthy volunteers and study groups are marked by an arrow (↑↓) and their color-matched p*-values.

**Figure 4** reveals shifts in the Treg subsets in healthy volunteers compared to KF, dialysis, and kidney transplant patients during the course of life. In healthy volunteers, CCR7^+^ RTE Tregs decreased (**Figure 4C**), while CCR7^+^ MN Tregs and CD31^-^ memory Tregs increased significantly (**Figures 4D, F**), proposing that with age, CCR7^+^ RTE Tregs differentiate *via* CD31^+^ memory Tregs into CD31^-^ memory Tregs without additionally consuming CCR7^+^ resting-MN Tregs. As neither CD31^+^ nor CD31^-^ TEMRA Tregs increased with age (**Figures 4A, B**), CCR7^+^ RTE Treg differentiation does not appear to be impaired. Similar age-dependent changes of the individual subsets were observed for all three patient groups except of CCR7^+^ MN Tregs in KF patients, which rather showed a decrease than an increase with age (**Figure 4D**), suggesting that in these patients CCR7^+^ MN Tregs are used to refill the CD31^-^ memory Treg pool with age.

However, significant age-independent changes in the composition of the total CD8^+^ Treg pool were observed for KF patients which correspond to those that occurred in healthy controls depending on age (**Figures 4C, D, F**). These data suggest an age-independently increased differentiation of CCR7^+^ RTE Tregs *via* CD31^+^ memory Tregs into CD31^-^ memory Tregs concomitant with the increased formation of CCR7^+^ resting-MN Tregs as naïve reserve population. In dialysis patients, an additional increase in the frequency of CD31^+^ memory Tregs and CD31^+^ TEMRA Tregs (**Figures 4A, E**) instead of CD31^-^ memory Tregs (**Figure 4F**) was observed, proposing reduced differentiation *via* this pathway in these patients.

In contrast, in transplant patients, CCR7^+^ RTE Tregs accumulated, while CD31^+^ memory Tregs and CD31^-^ memory Tregs declined strongly, and CD31^+^ TEMRA Tregs and CCR7^+^ MN Tregs remained increased (**Figures 4A, C–E**). This divergent distribution of CD8^+^ Treg subsets proposes fundamental differences in the Treg differentiation of these patients.

### Strong Accumulation of TEMRA Tresps in Kidney Transplant Patients

**Figure 5:**
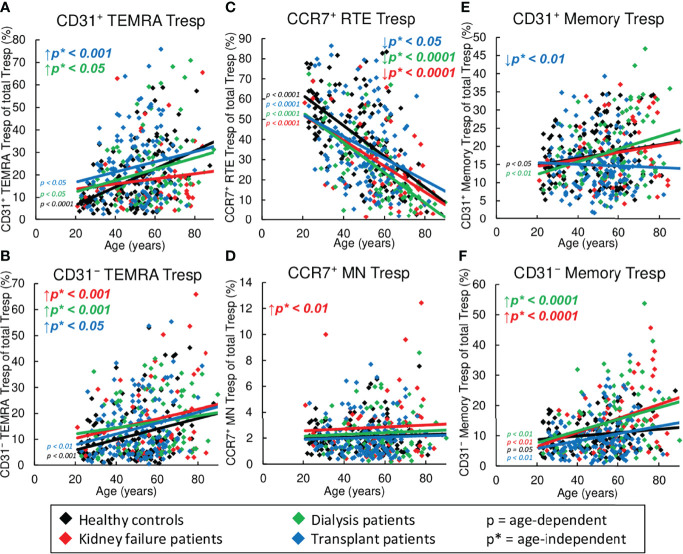
Changes in the composition of CD8^+^Tresps with differentially matured subsets in healthy controls (n=134), KF patients (n=57), dialysis patients (n=87), and kidney transplant patients (n=138). The figures present the percentages of CD31^+^ TEMRA **(A)**, CD31^-^ TEMRA **(B)**, CCR7^+^ RTE **(C)**, CCR7^+^ MN **(D)**, CD31^+^ memory **(E)**, and CD31^-^ memory Tresps **(F)** within total Tresps of healthy volunteers (♦), KF patients (♦), dialysis patients (♦), and kidney transplant patients (♦). Regression lines concerning changes in the percentages with age are shown for both healthy volunteers and all patient groups. Significant changes are marked by color-matched p-values. Age-independent significant differences between healthy volunteers and study groups are marked by an arrow (↑↓) and their color-matched p*-values.

**Figure 5** exhibits shifts in the Tresp subsets in healthy volunteers compared to KF, dialysis, and kidney transplant patients during the course of life. In healthy controls, CCR7^+^ RTE Tresps decreased with age (**Figure 5C**), while both CD31^+^ and CD31^-^ TEMRA Tresps, as well as CD31^+^ and CD31^-^ memory Tresps increased significantly (**Figures 5A, B, E, F**), suggesting diminished differentiation of CCR7^+^ RTE Tresps *via* CD31^+^ memory Tresps or direct proliferation into CD31^-^ memory Tresps with age. Since CCR7^+^ resting-MN Tresps did not accumulate (**Figure 5D**), continuous production and consumption of these cells seemed to ensure differentiation with age. Similar age-dependent changes of the individual subsets were observed for all three patient groups with the exception of CD31^+^ memory Tresps in transplant patients, which rather showed a decrease than an increase (**Figure 5E**), suggesting that there is a reduced production of CD31^+^ memory Tresps in these patients with age.

However, regardless of age, significantly decreased frequencies of CCR7^+^ RTE Tresps (**Figure 5C**), but increased frequencies of CD31^-^ TEMRA Tresps (**Figure 5B**) were seen for all patient groups, suggesting an impairment of CCR7^+^ RTE Tresp proliferation in all three patient groups. Thereby, KF patients additionally revealed increased frequencies of CCR7^+^ MN Tresps and CD31^-^ memory Tresps (**Figures 5D, F**), proposing an increased differentiation of CCR7^+^ RTE Tresp *via* CD31^+^ memory Tresps in which both CCR7^+^ resting-MN Tresps and CD31^-^ memory Tresps seem to arise. In dialysis patients, frequencies of CD31^+^ TEMRA Tresps instead of CCR7^+^ MN Tresps were increased (**Figures 5A, D**), proposing a shift in the differentiation in the direction of rather an increased consumption of CCR7^+^ resting-MN Tresps than differentiation *via* CD31^+^ memory Tresps. In transplant patients, frequencies of CD31^+^ and CD31^-^ TEMRA remained increased (**Figures 5A, B**), while frequencies of CD31^+^ memory Tresps were strongly decreased (**Figure 5E**). As frequencies of CD31^-^ memory Tresps were not increased in these patients (**Figure 5F**), a preferred differentiation of CCR7^+^ RTE Tresps into TEMRA Tresps may be assumed for these patients.

### Altered Release of Soluble Mediators From CD8^+^ Naïve, CM, and TEMRA, but Not EM Tresps in Kidney Transplant Patients

**Figure 6:**
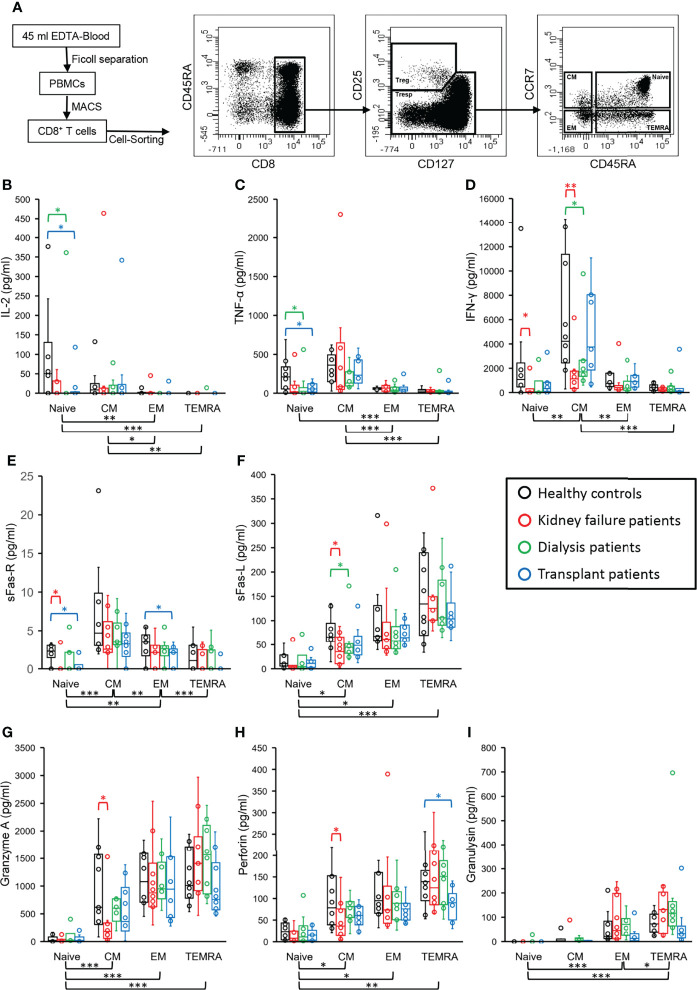
Functionality of naïve, CM, EM, and TEMRA Tresps in age-matched healthy volunteers (n=10), KF patients (n=10), dialysis patients (n=10), and kidney transplant patients (n=10). Total CD8^+^ T cells were isolated by MACS, stained with anti-CD8, anti-CD25, anti-CD127, anti-CD45RA and anti-CCR7 monoclonal antibodies to identify CD8^+^CD25^low+/-^CD127^+/-^ Tresps and sort them into CCR7^+^CD45RA^+^ naïve, CCR7^+^CD45RA^-^ CM, CCR7^-^CD45RA^-^ EM, and CCR7^-^CD45RA^+^ TEMRA Tresps **(A)**. After 72 hours of stimulation with anti-CD3/CD28 specific antibodies, bead-based analysis was performed to detect the concentrations of IL-2 **(B)**, TNF-α **(C)**, IFN-γ **(D)**, sFas-R **(E)**, sFas-L **(F)**, granzyme A **(G)**, perforin **(H)**, and granulysin **(I)** in the supernatants. Box plots represent the interquartile range with the median as a bar in black for healthy controls, red for KF patients, green for dialysis patients, and blue for kidney transplant patients. *p ≤ 0.05; **p < 0.01; ***p < 0.001.

To determine the influence of KF and subsequent kidney replacement therapies on the functionality of stimulated CD8^+^ Tresps, these cells were sorted into naïve, CM, EM, and TEMRA Tresps. Concentrations of IL-2, TNF-α, IFN-γ, sFas-R, sFas-L, granzyme A, perforin, and granulysin were determined in the supernatants of each subset in each of 10 age-matched healthy controls, KF, dialysis, and kidney transplant patients (**Figure 6**). In healthy volunteers, cytokines such as IL-2, TNF-α, and IFN-γ were mainly secreted by naïve and CM Tresps (**Figures 6B–D**). Concentrations of sFas-R were highest in the supernatants of CM and EM Tresps, while naïve and TEMRA Tresps showed weaker secretion (**Figure 6E**). Thus, it may be assumed that, compared to the other subsets, CM Tresps produce the largest amount of sFas-R and therefore, due to decreased membrane expression, may be less susceptible for Fas-L-induced apoptosis. Most sFas-L was detected in the supernatants of TEMRA Tresps, while moderate concentrations were determined in the supernatants of both CM and EM Tresps (**Figure 6F**), indicating that these cells express membrane-bound Fas-L rather than releasing it in soluble form. Cytotoxic mediators, such as granzyme A, perforin, and granulysin, were secreted by CM, EM, and most notably by TEMRA Tresps (**Figures 6G–I**).

In KF patients, IFN-γ secretion of naïve and CM Tresps was significantly reduced compared to healthy volunteers, whereas dialysis patients showed decreased secretion of IL-2 and TNF-α from naïve and IFN-γ from CM Tresps. After kidney transplantation, only naïve Tresps still showed decreased IL-2 and TNF-α secretion from CM Tresps while IFN-γ secretion from CM Tresps returned to normal levels (**Figures 6B–D**). Decreased sFas-R concentrations and therefore, presumably increased membrane-bound Fas receptor levels, occurred in all study groups in every cell subset. However, significance was only achieved in KF patients for naïve Tresps and in kidney transplant recipients for naïve and EM Tresps (**Figure 6E**). Compared to healthy controls, sFas-L concentrations also showed decreases in all patient groups, especially in KF and dialysis patients of CM Tresps, indicating that CM Tresps of these patient groups increased their cytotoxic capability *via* membrane-bound Fas-L-induced apoptosis (**Figure 6F**).

In contrast, secretion of cytotoxic mediators of CM Tresps, such as Granzyme A and perforin, was significantly decreased in KF patients but could be restored in dialysis and transplant patients (**Figures 6G, H**). However, in transplant patients, decreased levels of perforin, were detected in the supernatants of TEMRA Tresps compared with healthy controls (**Figure 6H**). These data may suggest that there is a possible impairment in the secretion of specific cytotoxic mediators in patients suffering from chronic kidney failure.

Due to the fact that the majority of the data analysis (**Figures 2**, **3**, **4**, **5**) is based on linear regression and the individual data points for each patient group are subject to wide variation, the R^2^ coefficients of all regression lines are shown in **Supplementary Table 1**.

## Discussion

**Figure 7:**
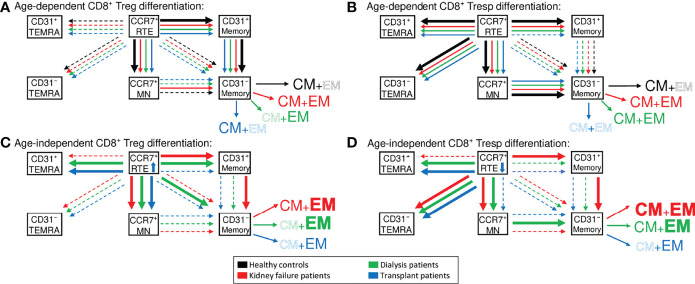
Proposed age-dependent differentiation pathways of CD8^+^ Tregs **(A)** and Tresps **(B)** in healthy controls (black), KF patients (red), dialysis patients (green), and kidney transplant patients (blue). Age-independently increased differentiation pathways are shown for CD8^+^ Tregs in **(C)** and Tresps in **(D)** for all patient groups compared to healthy volunteers.

T cell immunity was shown to be affected by both chronic KF and normal aging during the course of life (30). In this study, we demonstrate that in healthy volunteers CD8^+^ Tregs as well as CD8^+^ Tresps show age-dependent differences in the composition with differentially matured Treg/Tresp subsets. Therefore, our results indicate that the differentiation pathways with age may differ between cells. CCR7^+^ RTE Tregs differentiated *via* CD31^+^ memory Tregs into CD31^-^ memory Tregs, while CCR7^+^ RTE Tresps predominantly used CCR7^+^ resting-MN Tresps to replenish their CD31^-^ memory Tresp pool (**Figures 7A, B**). In contrast to CCR7^+^ RTE Tregs, which did not produce any TEMRA cells, most of CCR7^+^ RTE Tresps differentiated into both CD31^+^ and CD31^-^ TEMRA Tresps, which have probably lost most of their ability to further differentiate. These differences ensured increasing CD8^+^ Tregs, but decreasing CD8^+^ Tresps and therefore a significantly increasing CD8^+^ Treg/Tresp ratio with age in healthy controls. This was also observed in KF and dialysis patients, but could not be maintained in transplant patients. Transplant patients regained normal age-dependent CD8^+^ Treg/Tresp differentiation but could not increase total Tregs or their Treg/Tresp ratio with age, proposing an age-independently impairing effect of the immunosuppressive treatment on the age-dependent development of this ratio.

Accordingly, **Figure 7C** illustrates that frequencies of CCR7^+^ RTE Tregs were strongly increased in these patients, proposing a substantially inhibited differentiation of these cells. Especially CCR7^+^ RTE T cell differentiation *via* CD31^+^ memory T cells into CD31^-^ memory T cells appeared to be effectively blocked by the immunosuppressive therapy. Additionally, kidney failure comes along with a thymus involution and thus reduced distribution of CCR7^+^ RTEs (8). This dysfunction may be restored after kidney transplantation, which is another possible explanation for the markedly increased frequencies of CCR7^+^ RTE Tregs in kidney transplant patients. However, CCR7^+^ RTE Tresps were still decreased compared to healthy volunteers, so that the inhibition of the Tresp differentiation may be weaker than that of Tregs.

Independent of age, our data revealed an increased consumption of CCR7^+^ RTE Tregs/Tresps in KF and dialysis patients. Thereby, the increased differentiation of CCR7^+^ RTE Tregs/Tresps *via* CD31^+^ memory Tregs/Tresps resulted in the enhanced production of EM Tregs and both CM and EM Tresps in KF patients (**Figures 7C, D**). In dialysis patients, CCR7^+^ RTE Treg/Tresp differentiation *via* CD31^+^ memory Tregs/Tresps into CD31^-^ memory Tresps seemed to be decreased and replaced by direct CCR7^+^ Treg proliferation and increased consumption of CCR7^+^ resting-MN Tresps. This differentiation resulted in the increased formation of EM Tregs/Tresps (**Figures 7C, D**). Diminished CCR7^+^ RTE Treg/Tresp differentiation *via* CD31^+^ memory Tregs/Tresps into CD31^-^ memory Tregs/Tresps seemed to be indicated by the accumulation of CD31^+^ TEMRA Tregs/Tresps and was observed in both dialysis and transplant patients but not in KF patients. Such findings may explain why an intensification of the differentiation *via* CD31^+^ memory Tresps may no longer be possible in these patients. Diminished CCR7^+^ RTE Treg/Tresp proliferation seemed to be indicated by the accumulation of CD31^-^ TEMRA Tregs/Tresps. Compared to healthy controls, an accumulation of CD31^-^ TEMRA Tresps was observed in all patient groups, while CD31^-^ TEMRA Tregs could not be detected, indicating ongoing CCR7^+^ RTE Treg proliferation compared to reduced CCR7^+^ RTE Tresp proliferation.

However, these data also have limitations that make interpretation difficult. Although participant recruitment took place at a major transplant center, a sufficient number of patients could not be recruited to match the age distribution of the groups. This complicates data presentation and data analysis. Furthermore, the measurements reveal a high variance in all groups, indicating major differences of Treg/Tresp subset frequencies in the respective individuals (**Supplementary Table 1**). Kidney transplant patients revealed the highest variance in this study, presumably due to multiple clinical confounders. Those patients are characterized by a varying duration of dialysis therapy and thus exposure to uremic toxins. Another reason are the differing immunosuppressive approaches which also showed differences in their dosage. As transplant rejection episodes also come along with kidney dysfunction and high-dose immunosuppressive therapy, the number of previous rejections might also influence the frequencies of Treg/Tresp subsets. Additionally, it may be assumed that chronic viral infections after transplantation induce increased T cell differentiation and thus, alter Treg/Tresp subset distribution in these patients.

Previous studies considered an increased percentage of CD8^+^ TEMRA cells as a marker of T cell exhaustion, late graft dysfunction, and failure (31, 32), while others confirmed a positive correlation between exhausted PD1^+^CD57^-^ T cells and better allograft function (33). T cell-mediated rejection (TCMR) was shown to be associated with an increased percentage of CD8^+^ TEMRA cells (34), whereas exhausted CD8^+^CD28^-^ T cells lowered the risk for rejection (35, 36). In addition, Bottomley et al. showed that the percentage of exhausted CD8^+^CD57^+^ T cells is a predictor for future cutaneous squamous cell carcinoma (37). Such contradictory findings indicate that it may not be sufficient to assess total TEMRA cells, but at least the accumulation of TEMRA Tregs or Tresps should be evaluated differently. Future studies should also investigate whether exhaustion markers such as PD-1 and CD57 are expressed on CD31^+^ and CD31^-^ TEMRA Tregs/Tresps, or may provide evidence of immune senescence, detectable by loss of CD28 and expression of CD56, respectively. Thereby, our findings propose that the accumulation of CD31^+^ TEMRA Tregs may favor graft rejection in transplant patients as CD8^+^ Treg function may be impaired in these patients. Prospective studies could investigate whether the accumulation of CD8^+^ TEMRA Tregs correlates with an increased incidence of graft rejection. An immunosuppressive therapy matched to the percentage of CD8^+^ TEMRA Tregs may reduce the incidence of graft rejection. To what extent the accumulation of CD31^+^ or CD31^-^ TEMRA Tresps may influence the cytotoxic capacity of CD8^+^ Tresps needs further investigation.

**Figure 8:**
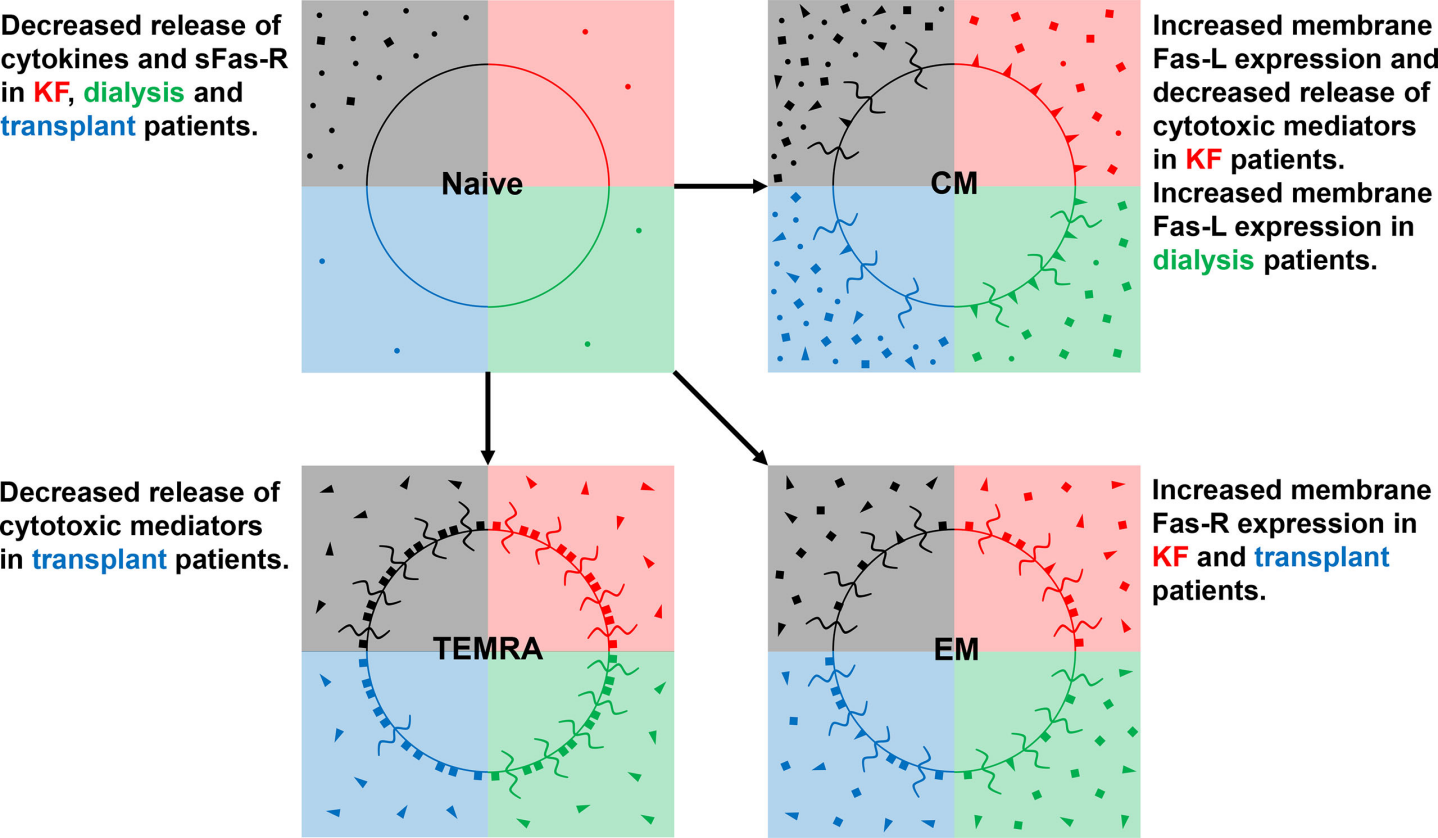
Differences in the release of cytokines (IL-2, TNF-α, and IFN-γ) (•), Fas-R (■), Fas-L (◄), and of cytotoxic mediators (granzyme A, perforin, and granulysin) (
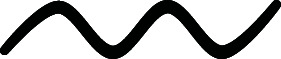
) between healthy controls (black quarter), KF patients (red quarter), dialysis patients (green quarter), and kidney transplant patients (blue quarter) in CD8^+^ naïve, CM, EM, and TEMRA Tresps.

**Figure 8** schematically illustrates our data concerning differences in the release of cytokines, sFas-R, sFas-L, and cytotoxic mediators of naïve, CM, EM, and TEMRA Tresps between healthy controls, KF, dialysis, and transplant patients. CM Tresps revealed the highest production of sFas-R and thus the lowest sensitivity for apoptosis. In KF patients, increased CM Tresp production was associated with the increased differentiation of CCR7^+^ RTE Tresps *via* CD31^+^ memory Tresps. These findings confirm our assumptions from previous studies that this differentiation pathway *via* CD31^+^ memory T cells favors the generation of CD31^-^ memory T cells with higher apoptosis resistance than the proliferation of CCR7^+^ RTE Tresps (38). This effect was already attenuated in dialysis patients and completely abolished in transplant patients, altering the differentiation of CCR7^+^ RTE Tresps toward increased proliferation into EM and TEMRA Tresps.

Our data suggest that CMs rather exerted cytotoxic effects through membrane-bound Fas-L expression, while TEMRAs showed the strongest secretion of cytotoxic mediators. EMs provided a hybrid of both mechanisms. Therefore, it may be assumed that EMs may arise by both the differentiation *via* CD31^+^ memory Tresps and proliferation. Increased CCR7^+^ RTE Tresp differentiation *via* CD31^+^ memory Tresps into CD31^-^ memory Tresps in KF patients was associated with the emergence of CM Tresps, which exhibited diminished secretion of both cytotoxic mediators and sFas-L compared to healthy controls. Presumably, this was instead associated with an increased membrane bound expression of Fas-L and therefore with an enhanced cytotoxic effect *via* Fas-L-induced apoptosis of these cells. Nevertheless, it remains doubtful whether the magnitude in the reduction of sFas-L secretion of about 30 pg/ml observed here can trigger such an effect. Otherwise, the decreasing CCR7^+^ RTE Tresp proliferation capacity in these patients may be associated with the formation of EM and TEMRA Tresps revealing higher apoptosis sensitivity by expressing high amounts of Fas-R, favoring effector functions *via* the release of cytotoxic mediators such as granzyme A, perforin, and granulysin. Hence, differentiation pathways and imbalances in the occurrence of Tresp effector subsets may play a crucial role in the efficacy of different cytotoxic mechanisms of CD8^+^ Tresps.

In kidney transplant recipients, TEMRA Tresps revealed a decreased release of perforin compared to healthy controls, which might be related to shifts in the ratio of CD31^+^ to CD31^-^ TEMRAs in these patients. Presumably, both TEMRA Tresp subsets have different abilities regarding the secretion of cytotoxic mediators. So far, TEMRAs were described as a heterogeneous cell population consisting of two subsets with a population aligning with naïve T cells and another one with a closer association to the effector memory subset (39). Moreover, the senescence marker CD57 seems to separate terminally differentiated CD57^+^ TEMRAs from CD57^-^ TEMRAs with longer telomeres, higher proliferative capacity, and differentiation plasticity (40). Otherwise, Rufer et al. described two TEMRA subsets of CD27^+^CD28^+^ and CD27^+^CD28^-^ cells with longer telomeres, higher levels of T cell receptor excision circles (TRECs), and intermediate cytolytic properties, suggesting that these subsets represent minor differentiated T cells with intermediate effector-like functions (41). Further studies need to examine whether CD31^+^ and CD31^-^ TEMRAs differ in their proliferation history, apoptotic stability, and cytotoxicity to better illuminate the heterogeneous composition of TEMRA cells.

In summary, our data show that normal age-dependent differentiation of CD8^+^ T cells differs between Tregs and Tresps since TEMRAs only arise in Tresps. Regardless of age, chronic KF caused significant shifts in the composition of the Treg/Tresp pool towards more mature Treg/Tresp subsets, indicating enhanced differentiation of CD8^+^ T cells in these patients. Notably, the appearance of CD8^+^ CM Tresps with enhanced Fas-L-mediated cytotoxicity but reduced Granzyme A and perforin secretion were induced. Dialysis patients may be able to largely maintain the emergence of such CM Tresps, while transplant patients mainly enriched TEMRA Tresps that produced decreased amounts of perforin. In summary, our data may propose that the immunosuppressive therapy in transplant patients inhibits the differentiation of CD8^+^ Tregs more than that of CD8^+^ Tresps. Therefore, CD8^+^ Tregs are more likely to be able to expand on demand than CD8^+^ Tresps, so these patients may have an increased susceptibility to viral infections and malignancies but a lower risk of TCMR.

## Data Availability Statement

The raw data supporting the conclusions of this article will be made available by the authors, without undue reservation.

## Ethics Statement

The studies involving human participants were reviewed and approved by Ethics Committee of the Medical Faculty of Heidelberg, University of Heidelberg, Heidelberg, Germany, reference number (S-523/2012/05.07.2018).

## Author Contributions

JL, MS and AS designed the study. JL performed the study. VE provided technical support regarding the cell sorting. MS and FK coordinated the sample collection. MZ contributed important patients. JL, MS and AS analyzed the data and wrote the manuscript. All authors contributed to the final version of the manuscript and approved it.

## Funding

This work was supported by a Kidney Center Heidelberg research grant. The project was financed with funds from the research budget of the Department of Nephrology, University of Heidelberg, Germany.

## Conflict of Interest

The authors declare that the research was conducted in the absence of any commercial or financial relationships that could be construed as a potential conflict of interest.

## Publisher’s Note

All claims expressed in this article are solely those of the authors and do not necessarily represent those of their affiliated organizations, or those of the publisher, the editors and the reviewers. Any product that may be evaluated in this article, or claim that may be made by its manufacturer, is not guaranteed or endorsed by the publisher.

## Supplementary Material

Supplementary Table 1 R^2^ coefficients of determination of the linear regressions. CM, central memory; EM, effector memory; KF, kidney failure; MN, mature naïve; RTE, recent thymic emigrant; TEMRA, terminally differentiated effector memory; Treg, regulatory T cell; Tresp, responder T cell.
