# Supplementary material for: Chronic Kidney Failure Provokes the Enrichment of Terminally Differentiated CD8+ T Cells, Impairing Cytotoxic Mechanisms After Kidney Transplantation
Source: Front Immunol. 2022 May 3;13:752570. doi: 10.3389/fimmu.2022.752570 (PMC9110814; doi:10.3389/fimmu.2022.752570)
Supplement: Supplementary Table 1 — R2 coefficients of determination of the linear regressions. CM, central memory; EM, effector memory; KF, kidney failure; MN, mature naïve; RTE, recent thymic emigrant; TEMRA, terminally differentiated effector memory; Treg, regulatory T cell; Tresp, responder T cell. [file Table_1.docx]

| **Supplementary Table 1:** R^2^ coefficients of determination of the linear regressions. | | | | |
| --- | --- | --- | --- | --- |
|  | **Healthy controls** | **KF patients** | **Dialysis patients** | **Transplant patients** |
| Figure 2 |  |  |  |  |
| (A) CD8^+^ T cells | 0.1095 | 0.0203 | 0.0188 | 0.0188 |
| (B) CD8^+^ Treg | 0.0578 | 0.0761 | 0.0013 | 0.0013 |
| (C) CD8^+^ Tresp | 0.0578 | 0.0761 | 0.0013 | 0.0013 |
| (D) CD8^+^ Treg/Tresp ratio | 0.0569 | 0.0741 | 0.0015 | 0.0015 |
| Figure 3 |  |  |  |  |
| (A) Naïve Treg | 0.2867 | 0.2287 | 0.2293 | 0.0884 |
| (B) CM Treg | 0.1908 | 0.0880 | 0.0310 | 0.0653 |
| (C) TEMRA Treg | 0.0001 | 0.0705 | 0.0002 | 0.0010 |
| (D) EM Treg | 0.0109 | 0.0865 | 0.1050 | 0.0102 |
| (E) Naïve Tresp | 0.3459 | 0.2791 | 0.3928 | 0.1227 |
| (F) CM Tresp | 0.0703 | 0.0664 | 0.0803 | 0.0114 |
| (G) TEMRA Tresp | 0.2533 | 0.0551 | 0.0833 | 0.0797 |
| (H) EM Tresp | 0.0065 | 0.0810 | 0.0518 | 0.0048 |
| Figure 4 |  |  |  |  |
| (A) CD31^+^ TEMRA Treg | 0.0268 | 0.0776 | 0.0035 | 0.0001 |
| (B) CD31^-^ TEMRA Treg | 0.0005 | 0.0144 | 0.0028 | 0.0028 |
| (C) CCR7^+^ RTE Treg | 0.3743 | 0.2511 | 0.3695 | 0.1239 |
| (D) CCR7^+^ MN Treg | 0.0481 | 0.0181 | 0.0296 | 0.0278 |
| (E) CD31^+^ memory Treg | 0.0078 | 0.0203 | 0.0011 | 0.0032 |
| (F) CD31^-^ memory Treg | 0.1667 | 0.2358 | 0.1246 | 0.0734 |
| Figure 5 |  |  |  |  |
| (A) CD31^+^ TEMRA Tresp | 0.2430 | 0.0166 | 0.0602 | 0.0394 |
| (B) CD31^-^ TEMRA Tresp | 0.0939 | 0.0421 | 0.0249 | 0.0592 |
| (C) CCR7^+^ RTE Tresp | 0.3493 | 0.2936 | 0.4054 | 0.1280 |
| (D) CCR7^+^ MN Tresp | 0.0041 | 0.0025 | 0.0039 | 0.0017 |
| (E) CD31^+^ memory Tresp | 0.0346 | 0.0314 | 0.0804 | 0.0015 |
| (F) CD31^-^ memory Tresp | 0.0281 | 0.1420 | 0.0821 | 0.0550 |
| *CM, central memory; EM, effector memory; KF, kidney failure; MN, mature naïve; TEMRA, terminally differentiated effector memory; Treg, regulatory T cell; Tresp, responder T cell* | | | | |
